# Channel Measurement and Feasibility Test for Wireless Avionics Intra-Communications

**DOI:** 10.3390/s19061294

**Published:** 2019-03-14

**Authors:** Inkyu Bang, Hyunwoo Nam, Woohyuk Chang, Taehoon Kim, Jong-Myung Woo, Choul-Young Kim, Tae-Won Ban, Pangun Park, Bang Chul Jung

**Affiliations:** 1 Department of Computer Science, School of Computing, National University of Singapore, Singapore 117417, Singapore; inkyu@comp.nus.edu.sg; 2 School of Electrical Engineering, College of Engineering, KAIST, Daejeon 34141, Korea; hw.nam@kaist.ac.kr; 3 Agency for Defense Development, Daejeon 34186, Korea; whchang@add.re.kr (W.C.); taehoonkim@add.re.kr (T.K.); 4 Department of Electronics Engineering, Chungnam National University, Daejeon 34134, Korea; jmwoo@cnu.ac.kr (J.-M.W.); cykim@cnu.ac.kr (C.-Y.K.); pgpark@cnu.ac.kr (P.P.); 5 Department of Information and Communication Engineering, Gyeongsang National University, Tongyeong 53064, Korea

**Keywords:** channel measurements, IEEE 802.11, monopole antennas, sensor networks, software-defined radio (SDR), wireless avionics intra-communications (WAIC)

## Abstract

Wireless avionics intra-communication (WAIC) refers to a wireless communication system among electronic components (e.g., sensors and actuators) that are integrated or installed in an aircraft and it is proposed to replace heavy and expensive wired communication cables. Recently, the use of a frequency band (4.2–4.4 GHz) for the WAIC (so-called, WAIC band) has been approved by international telecommunication union (ITU). Accordingly, several existing wireless protocols such as IEEE 802.11 and IEEE 802.15 are being considered as candidate techniques for the intra-avionics sensor network. In this paper, we perform a real field experiment to investigate wireless channel characteristics in intra-avionics sensor networks at the WAIC bands by a software-defined radio platform (universal software radio peripheral, USRP) and self-produced monopole antennas for the WAIC band. Through the experiment, we validated the feasibility of IEEE 802.11 protocol for the intra-avionics sensor network at the WAIC band in real aircraft environments. Furthermore, based on the measurement data, we evaluated the bit error rate (BER) performance of multiple antenna techniques where we considered the maximum ratio combining (MRC) for the multi-antenna receiver and the space-time block coding (STBC) for the multi-antenna transmitter.

## 1. Introduction

Wireless avionics intra-communications (WAIC) refers to radio communication systems that interconnect avionics components (e.g., sensors and actuators) installed in an aircraft [1]. Wireless technologies to enable communications outside an aircraft have been broadly investigated and developed to support various necessary functionalities such as aircraft-ground control, inter-aircraft communications, and radar. Contrary to this, applying wireless networks (e.g., intra-avionics sensor networks) inside the aircraft has been relatively less highlighted due to the concerns about wireless communication on reliability, safety, and security, compared to wired ones [2]. However, recent advances in wireless communication technologies show that mandatory operational-criteria (e.g., reliability and robustness) of the aircraft can be sufficiently satisfied through wireless networks, which results in the overall weight reduction of the aircraft (e.g., removing the wired cables) [3]. In particular, statistics show that wireless networks will lead to 15% lighter designs than existing ones in terms of overall weight and will bring 12% increase in fuel efficiency in the aircrafts [4]. Accordingly, international telecommunication union (ITU) decided to allocate a frequency band from 4.2 GHz to 4.4 GHz for the WAIC at world radio-communication conference (WRC) 2015 [5].

Several studies related to WAIC are reported recently. Raharya and Suryanegara investigated the compatibility of WAIC systems with radio altimeters at 4.2–4.4 GHz by simulations [6], and analyzed the modulation performance in the WAIC systems with consideration of various factors such as modulation efficiency, net average application data rate, and protocol overhead [7]. Sámano-Robles et al. [2] investigated design issues in WAIC systems across several layers: from physical-layer to application layer. Park and Chang [8] proposed a mathematical framework to evaluate wireless protocol candidates in terms of the deadline missing probability and the packet loss probability. Sekiguchi et al. [9] estimated path loss between inside a passenger aircraft (Airbus 320-200) cabin to exterior near the tip of the main wing. Although various aspects of WAIC have been thoroughly investigated in previous work, the feasibility test on wireless protocol candidates of WAIC has not been explicitly validated via real-field experiments. To investigate the feasibility of wireless protocols, channel measurement should be preliminarily performed before the planning and design of any wireless systems.

In this study, we performed a real-field experiment to measure channel characteristics of the WAIC bands (4.2–4.4 GHz) and to investigate the feasibility of IEEE 802.11 protocol in real aircraft environments. (IEEE 802.11 physical-layer specification is one of the most promising protocols for the high data-rate applications in WAIC systems [3]. In addition, we considered KAI KT-1 Woongbi (http://www.koreaaero.com/english/product/fixedwing_kt-1.asp) for our experiment.) Further, reliable transmission techniques (e.g., maximum ratio combining and space-time block coding) in intra-avionics sensor network environments were evaluated using our measured data. (The notion of WAIC includes intra-avionics sensor networks. However, in this paper, we use the terms WAIC and intra-avionics sensor networks interchangeably to indicate applying wireless networks in the aircraft.) We believe that our field test shed light on the first step for implementing WAIC systems in practice. The main contributions of this paper are summarized as follows:

- We used software-defined radio devices and modified the IEEE 802.11 application framework to measure wireless channel for WAIC systems (see Section 2 and Section 3).
- Commercial antennas do not support the WAIC bands (4.2–4.4 GHz), and thus we self-produced monopole antennas to transmit and receive wireless signals at the WAIC bands (see Section 3).
- We performed point-to-point wireless channel measurements and feasibility test of adopting IEEE 802.11 protocol in a real aircraft (KAI KT-1 Woongbi). (Specific reasons why we chose KAI KT-1 Woongbi as our test aircraft are explained in Section 3.) We obtained a power delay profile (PDP) and a root mean square (RMS) delay between measured points (see Section 3 and Section 4).
- We simulated the bit error rate (BER) performance using our measurement data when we considered a candidate sensor network scenario and reliable transmission techniques such as a maximum ratio combining (MRC) and space-time block coding (STBC) schemes (see Section 5).

## 2. Overview of IEEE 802.11 Physical Layer

In this section, we briefly introduce an orthogonal frequency division multiplexing (OFDM) based physical layer specification in IEEE 802.11 protocols and its channel estimation principle.

### 2.1. IEEE 802.11 Physical Layer and OFDM

IEEE 802.11 is a set of media access control layer and physical layer protocol specifications for implementing wireless local area network (WLAN) in the industrial, scientific and medical (ISM) frequency bands (usually, 2.4 and 5 GHz). The original version of the standard was released in 1997 (IEEE 802.11-1997 or legacy) and subsequent amendments (IEEE 802.11a IEEE 802.11b, IEEE 802.11g, IEEE 802.11n, IEEE 802.11ac, etc.) have been done to support various system requirements. In most releases of IEEE 802.11 protocols, OFDM was widely adopted due to its robustness on the multi-path fading and its flexibility on operating bandwidth. Table 1 shows OFDM parameters used for most of IEEE 802.11 protocols.

Note that high throughput or very high throughput modes with different OFDM configurations are specified and provided in recent releases (e.g., IEEE 802.11n or IEEE 802.11ac). However, legacy mode based on Table 1 is mandatory due to backward compatibility and we mainly considered this legacy mode during our experiment.

### 2.2. Channel Estimation

**Figure 1:**
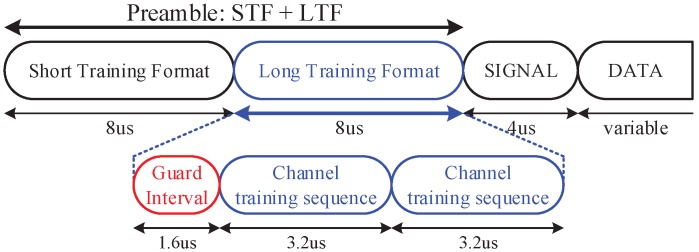
OFDM frame structure in IEEE 802.11 [10].

Figure 1 shows the basic OFDM frame structure used in IEEE 802.11 protocol which consists of a preamble, a SIGNAL, and multiple data fields. Specifically, the preamble field contains two types of training formats: short training format (STF) and long training format (LTF). STF is used to detect the beginning of OFDM frame and to obtain the timing synchronization, and LTF is used for channel estimation in frequency domain. LTF consists of two OFDM symbols (3.2×2=6.4μs) with guard interval (0.8×2=1.6μs) and is used to repeatedly carry a pre-defined long training symbol.

The long training symbol consists of 53 subcarriers including the zero value at DC, which are modulated by the elements of the sequence *L*, given by [10]

(1) $$\begin{array}{cc} L_{-26,26}= & \left\{1,1,-1,-1,1,1,-1,1,-1,1,1,1,1,1,1,-1,-1,1,1,-1,1,-1,1,1,1,1,0,\right.\\ & \left.\;\;1,-1,-1,1,1,-1,1,-1,1,-1,-1,-1,-1,-1,1,1,-1,-1,1,-1,1,-1,1,1,1,1\right\}, \end{array}$$

where the subscript *k* indicates subcarrier index, i.e., $k\in \left[-26,+26\right]$.

The long training symbol generated based on $L_{-26,26}$ in Equation (1) is repeated over two OFDM symbols to improve channel estimation accuracy. Thus, the channel response in frequency domain can be estimated as follows:

(2) $$\begin{array}{c} \hat{H}\left(k\right)=L_{k}Y\left(k\right), \end{array}$$

where Y(k) denotes the received signal at *k*th subcarrier, which is transformed from the received preamble in time domain. Using IFFT (Inverse Fast Fourier Transformation) operation, channel response in time domain (equivalently, delay domain) can be obtained as follows:

(3) $$\begin{array}{c} \hat{h}\left(\tau \right)=\sum_{k=-26}^{+26}\hat{H}\left(k\right)\exp\left(j2\pi k\Delta_{F}\right), \end{array}$$

where $\hat{H}\left(k\right)$ in Equation (2) and $\Delta_{F}$ is defined in Table 1, respectively. Note that we can measure wireless channel for WAIC systems by using IEEE 802.11 OFDM preambles.

## 3. Experimental Setup

**Figure 2:**
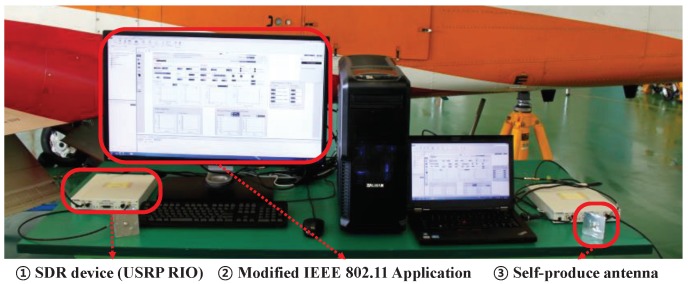
Experimental setup.

In this section, we introduce the details of the experimental setup including software, hardware, and measurement environment. An overview of major components (hardware and software) in our experimental setup is described in Figure 2.

### 3.1. Hardware and Software

We used the software-defined radio device (USRP RIO by National Instrument (http://www.ni.com/en-us.html), ① in Figure 2) to apply IEEE 802.11 physical layer specifications into WAIC bands (4.2–4.4 GHz).To measure wireless channel in WAIC bands, we ran our software, which was newly modified from IEEE 802.11 application framework (commercially provided by National Instrument) based on a visual programming language called LabVIEW (② in Figure 2). Although we used commercial software, open-source codes based on GNU radio are also available in the case of an experiment under limited budget (http://github.com/bastibl/gr-ieee802-11). We used two USRPs: one for transmitter only with transmit antenna and the other for receiver only with receive antenna.

### 3.2. Monopole Antenna for 4.2–4.4 GHz

As mentioned in Section 1, commercial antennas do not support 4.2–4.4 GHz bands since those are allocated for WAIC systems by ITU. For example, VERT 2450 antenna, commonly used with SDR device, only supports dual-band (2.4–2.5 GHz and 4.9–5.9 GHz) for Wi-Fi. Thus, we self-produced monopole antennas for our experiment (③ in Figure 2), which operate in WAIC bands.

**Figure 3:**
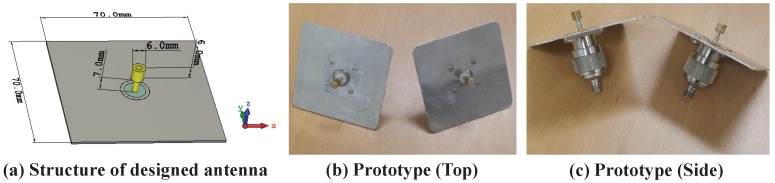
Structure and prototype of the designed monopole antenna.

Figure 3 shows an overall structure of designed monopole antenna and its prototype. We prepared two prototype antenna modules and measured a single transmit–receive link by separately connecting them to two USRPs. Before the feasibility test, we first measured the scattering parameter (*S*-parameter) and the radiation beam pattern to verify the performance of the designed monopole antenna.

*S*-parameters are used to describe the electrical behavior of linear electrical networks [11]. In general, antenna matching is described as a simple two-port network and $S_{11}$ parameter indicates the input power reflection coefficient. Thus, $S_{11}$ value should be low to radiate input signals to the air.

The radiation beam pattern indicates the directional (angular) dependence of the strength of the radio waves from the antenna [12]. In general, the relative amplitude normalized to the total radiated power is plotted, separately in the electric field plane (*E*-plane) and the magnetic field plane (*H*-plane).

**Figure 4:**
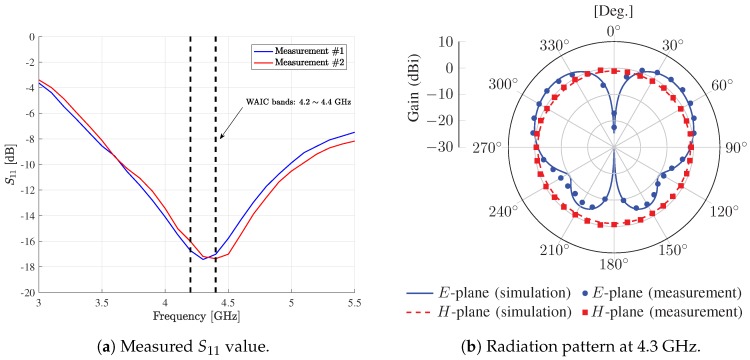
Measured performance of the designed monopole antenna.

Figure 4 shows the measured performance of the designed monopole antenna. Figure 4a shows measured $S_{11}$ values for varying operating frequency. For each measurement, low $S_{11}$ values are observed at WAIC bands (−17.42 and −17.19 dB at 4.3 GHz), which indicates that input power fed to transmit antenna is well radiated into the air, approximately 98% of input signal. Figure 4b shows the radiation beam patterns at 4.3 GHz. xy-plane and yz-plane at the designed antenna indicates *E*-plane and *H*-plane, respectively. Measurement results agreed well with simulation ones. Additionally, the maximum antenna gain aws 3.89 dBi and half-power bandwidth (HPBW) at *E*-plane was about 60°. Note that, based on measurements, we verified that the designed monopole antenna was appropriate to transmit and receive signals at WAIC bands.

### 3.3. Measurement Environment

**Figure 5:**
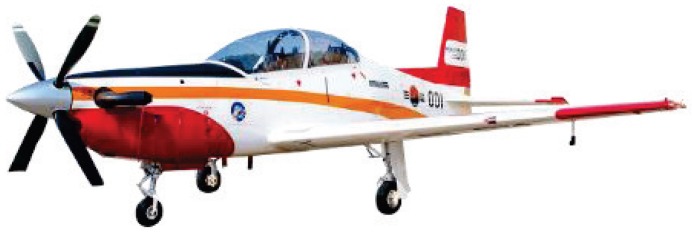
The appearance of KT-1 Woongbi.

The main objectives of our experiment were to measure wireless channel and to test the feasibility of adopting IEEE 802.11 physical layer specification in the real aircraft. We performed our experiment in a flight test site located in South Korea. The target aircraft was KT-1 Woongbi, shown in Figure 5, which is used for basic training aircraft in Korean, Indonesian, Turkish, and Peruvian Air Forces.

WAIC is designed to reduce the use of cable, eventually resulting in the weight of a big-sized aircraft and saving fuel. However, it is worth noting that the basic cover material for both KT-1 Woongbi and a WAIC-targeted aircraft such as a passenger plane (e.g., airbus A380) would be similar, so-called *poly-fiber* [13]. From the perspective of the wireless communication, a major difference between wireless networks in the big-sized aircraft and the small-sized one would be the number of multi-hops including relay nodes. Thus, there are two advantages of doing the field test on the small-sized aircraft such as KT-1 Woongbi: (1) Our experiment implies all feasibility tests based on in a single-hop network, which could be a part of a local network in the big-sized aircraft. (2) Our approach provides clear intuitions on the feasibility of WAIC by analyzing the small-sized aircraft such as a module of the big-sized aircraft, instead of a relatively complicated experiment in the big-sized aircraft. (Since our experiment on the small-sized aircraft can be considered as a module test of the big-sized aircraft, a combining test should be done (i.e., combining multiple single hop networks to one multi-hop network. However, this is beyond the scope of this paper.) The specification of this aircraft is briefly summarized in Table 2 [14].

**Figure 6:**
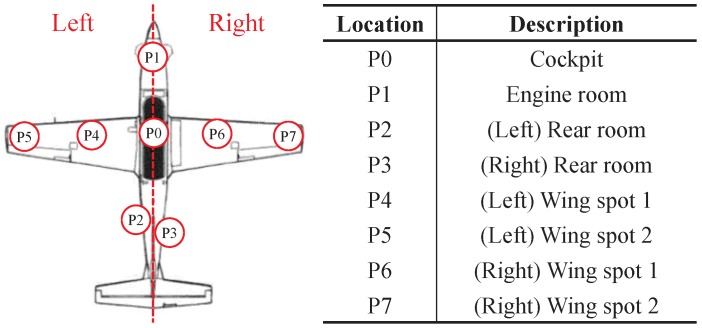
Measurement points in KT-1 Woongbi and their descriptions.

Specifically, we set several measurement points in KT-1 Woongbi for our experiment, as described in Figure 6. At each point, we obtained multiple measurement samples to remove the effect of white noise based on time (or frequency) domain averaging. Additionally, we performed measurements by changing spatial positions in one point (e.g., P1) for spatial averaging. Note that a distance between measurement points is specified in Section 4. Please refer to Appendix A for inside photos of KT-1 Woongbi at each measurement point.

**Figure 7:**
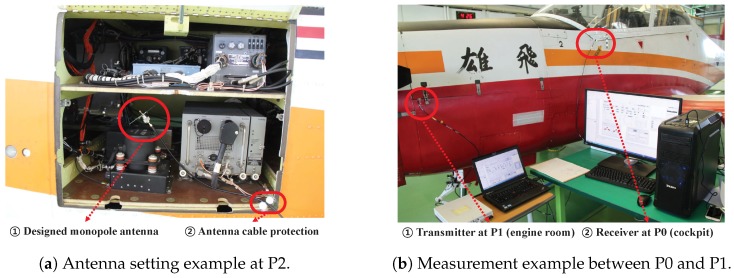
Measurement example.

Figure 7 shows measurement example in our experiment including antenna setup example. Figure 7a depicts an example of setting the monopole antenna at measurement point P2 (left rear room). Note that we put the antenna at the desired point after open the cover, and obtained measurement results after closing the cover. To properly close the cover and prevent damage to the antenna cable, we set small materials (e.g., sphere-type styrofoam, ② in Figure 7a) at the corner of the cover closure. Figure 7b shows an overall measurement setup between P0 (cockpit) and P1 (engine room) after the cover is closed.

During our experiment, we measured a *single* transmitter–receiver link at *one time* instead of multiple links simultaneously. (If we measured multiple links simultaneously at the same frequency band, channel measurement could be distorted by interference between multiple links.) Obviously, there was interference when we considered multiple data transmission at the same time (e.g., transmission of measured data by multiple sensors). To resolve the interference issue during data transmissions, we used conventional TDMA (time-division multiple access) as in [8].

## 4. Measurement Results

In this section, we introduce data processing methods for raw data obtained in our experiment, and the measurement results.

### 4.1. Data Processing

As described in Section 2, channel estimation in IEEE 802.11 physical layer was performed in frequency domain. Thus, measured samples in our experiment were also obtained in frequency domain, which is represented as follows:

(4) $$H\left(f,l,n\right),$$

where *f*, *l*, and *n* denote frequency (equivalently, subcarrier index), location index, and sample index, respectively. Location index indicates the different locations in the same measurement point, described in Figure 6, to obtain spatial averaging results.

We obtained 100 samples per each measurement and average them to reduce the effect of white noise (this could be possible if we obtained samples within channel coherence time; since our experiment was performed under controlled environment, it was applicable in our situation), given by

(5) $$\bar{H}\left(f,l\right)=\sum_{n=1}^{100}H\left(f,l,n\right).$$

The channel impulse response, $h\left(\tau ,l\right)$, can be obtained by applying IFFT on $\bar{H}\left(f,l\right)$ in Equation (5). Accordingly, the instantaneous power delay profile (PDP) and the averaged PDP (APDP) are calculated, respectively, as follows [15]:

(6) $$\begin{array}{cc} PDP\left(\tau ,l\right) & ={\left|h\left(f,l\right)\right|}^{2},\\ APDP\left(\tau \right) & =\frac{1}{\left|L\right|}\sum_{l\in L}PDP\left(\tau ,l\right), \end{array}$$

where L denotes a set of different locations in a measurement point in Figure 6. Additional useful metrics such as mean excess delay ($\bar{\tau }$) and root mean square (RMS) delay spread ($\sigma_{\tau }$) are calculated based on APDP and defined as follows [16]:

(7) $$\begin{array}{cc} \bar{\tau } & =\frac{\int_{-\infty }^{\infty }APDP\left(\tau \right)\tau d\tau }{\int_{-\infty }^{\infty }APDP\left(\tau \right)d\tau },\\ \sigma_{\tau } & =\sqrt{\frac{\int_{-\infty }^{\infty }APDP\left(\tau \right)\tau^{2}d\tau }{\int_{-\infty }^{\infty }APDP\left(\tau \right)d\tau }-{\bar{\tau }}^{2}}. \end{array}$$

### 4.2. Feasibility Test and Power Delay Profile

To verify the feasibility of adopting IEEE 802.11 physical layer specification in KT-1 Woongbi, we checked block error rate (BLER), constellation, and throughput during packet transmissions. During our experiments, we set a transmit power of USRP as 20 dBm (=100 mW). Note that the transmit power set in our experiment can be a reference value for the minimum required power for the communication in the aircraft since SDR devices (i.e., USRPs) usually have less performance in terms of radio transmission/reception compared to commercial radio devices. (It would be interesting to see the performance result (i.e., transmission success/fail) with commercial grade radio devices. However, unfortunately, as mentioned in Section 3, there are no commercial antennas that support those band since the 4.2–4.4 GHz bands are allocated for WAIC by ITU. Thus, we only considered the results measured by the self-produced monopole antennas).

**Figure 8:**
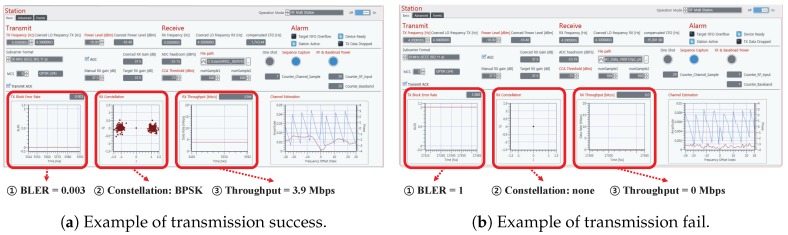
Feasibility test.

Figure 8 shows feasibility test in our experiment. Figure 8a indicates success of packet transmission, determined by almost zero BLER, clear constellation points, and a certain level of throughput. On the other hand, Figure 8b represents fail of packet transmission, which shows 100% BLER, none constellation points, and zero throughput. Based on the feasibility test, we classified the measurement points in Figure 6 into the communication regions summarized in Table 3. The criterion of classification was the success/fail of packet transmissions between the given measurement points. Fortunately, we could clearly differentiate the success/fail of packet transmissions, as shown in Figure 8, between any measurement points. Specifically, we chose any two measurement points among P0–P7. If a packet transmission was successful (i.e., zero BER, clear constellation points) between those two points, then those two points were considered as the same communication region. In other words, in our context, communication regions mean an area where reliable wireless packet transmissions are feasible between any points in each region under the transmit power constraint (≤20 dBm). (The power constraint under 20 dBm was sufficient in our experiments since we considered intra-avionics sensor network environments.) For example, reliable wireless packet transmissions were feasible between P0 and P2 since they were in the same Region 2. However, if we considered P0 (Region 1 and 2) and P7 (Region 4), then we would experience a transmission failure.

**Figure 9:**
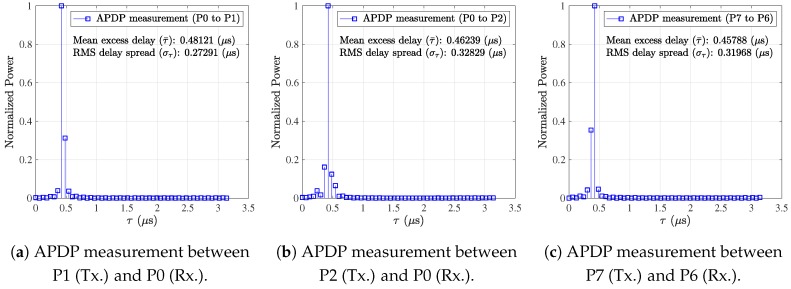
APDP results at various locations (20 MHz at 4.3 GHz).

Figure 9 shows APDP measurement results at each communication region. We omit the result for Region 3 (left wing) in Table 3 since it was similar to that in Region 4 (right wing). Since we used OFDM transmission in 20 MHz (effectively, 52 subcarriers with 16.6 MHz) at 4.3 GHz, we had about 0.06 μs $\left(=\frac{1}{16.6\;\mathrm{MHz}}\right)$ time resolution in our measurements. For all measurements, line-of-sight (LOS) signal path was a dominant component of measured APDP since the measurement distances were relatively short (less than 2 m). We notice that the engine room (P1) contained more electrical devices and wires compared to the left rear room (P2), and those contributed to more reflections (please refer to Appendix A for detailed inside photos). Accordingly, Figure 9a shows a relatively clear non-LOS (NLOS) effect (i.e., multiple prominent peaks) rather than Figure 9b. Although NLOS effects were observed in each measurement, those were not a crucial factor for implementing wireless networks in this environment.

**Figure 10:**
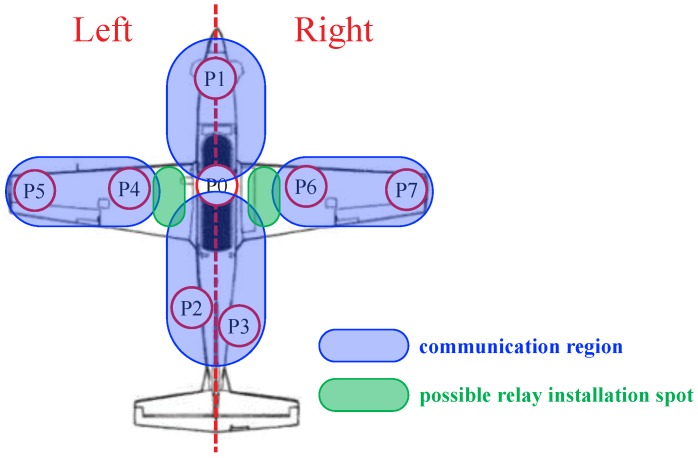
Communication regions in KT-1 Woongbi and an example of possible relay installation spots.

**Remark** **1.** *It is worth noting that we performed our experiment in both LOS and NLOS environments where electronic devices and mechanic components are filled in the measurement points, as shown in Figure 7a. Interestingly, our measurement results properly fit a single tap channel model rather than multi-tap channel model due to the dominant LOS channel effect rather than the NLOS one. Accordingly, our measurement results and feasibility test provided insight for WAIC system design. Although many studies assume Rayleigh fading channel environment, LOS channel effect has to be taken into account if we consider WAIC system where short-distance wireless transmission is preferred, as in our experiment. As we discussed, communication regions of intra-avionic sensors are limited based on their maximum transmit power. Accordingly, relay nodes have to be carefully installed at the intersection of communication regions (Table 3) in order to guarantee reliable wireless communications in the entire aircraft area. For example, intersection points between the main body and right/left wings are a proper place for relay nodes, as shown in Figure 10. (Note that the relay installation would mainly be determined by the targeted aircraft structure.) Additionally, we can utilize one of the existing interference mitigation/spatial diversity techniques at relay nodes to guarantee QoS (quality of service) [17,18,19].*

### 4.3. Path Loss Measurement

We measured path loss in KT-1 Woongbi by considering transmit/receive power, antenna cable loss, and the designed monopole antenna gain. To verify our measurement results, we also considered formally verified radio propagation simulation, carried out by the international aerospace community [3]. The simulations in [3] were classified into six groups as follows: (A) intra-cab in and intra-flight deck; (B) Inter-Cabin; (C) Inter-Cabin-to-Lower Lobe and Inter Cabin-to-Flight Deck; (D) Inter-Cabin-to-Exterior; (E) Inter-Cabin-to-Landing Gear and Inter-Lower-Lobe to Exterior; and (F) Inter-Exterior.

**Figure 11:**
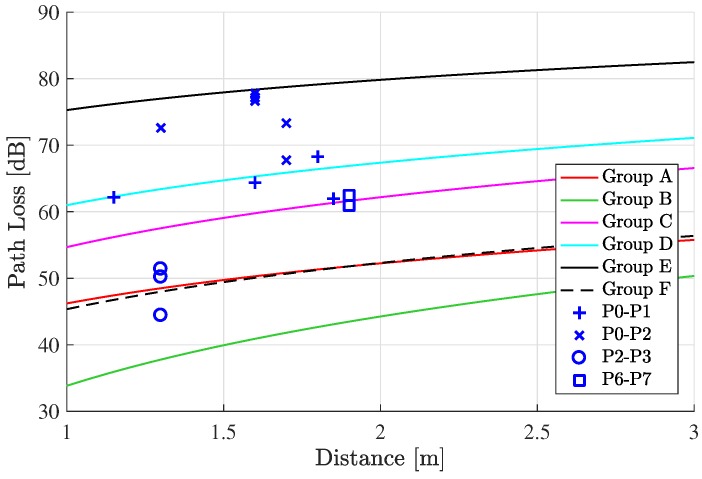
Path loss measurements at 4.3 GHz.

Figure 11 shows path loss measurements at 4.3 GHz. There is some variance among results at the same measurement point (e.g., P2–P3) due to hardware limitation and dynamic ranges of our measurement system. However, most of our measurement results range within those of Groups A–F. In particular, some measurements (e.g., P0–P1) showed similar path loss values with the results in [3] (e.g., Group D), which indicates the validity of our measurements.

**Remark** **2.** *We obtained at most 1.9 m transmit range under the power constraint (i.e., 100 mW) in our test, as shown in Figure 11. One might think the transmission range is short. However, note that our experiment was done based on SDR devices (i.e., USRPs), which usually have less performance than commercial radio devices. Thus, the transmission range would not be an issue if we used commercial radio devices. Additionally, if the short transmission issue happened due to the path loss characteristic of the aircraft, then we could utilize relay nodes to overcome the short transmission range, as discussed in Remark 1.*

## 5. Simulations with Measured Data

We simulated the bit error rate (BER) performance using our measurement data when we considered a candidate sensor network scenario for applying wireless networks in the aircraft such as intra-avionics sensor networks. Note that we utilized channel data samples measured in all measurement points during the experiment for simulations.

### 5.1. Simulation Environments

**Figure 12:**
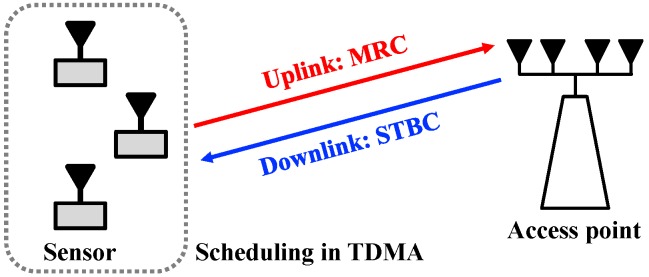
The wireless network model considered during simulation (e.g., the number of sensors: 3).

For simulations, we considered a sensor network that consists of multiple sensors and a single access point (AP) as described in Figure 12. We assumed that each sensor and the AP were equipped with a single and *M* antennas, respectively. We considered the TDMA in a sensor node scheduling to avoid interference issue and guarantee the reliability of wireless link. For modulations, we considered quadrature phase-shift keying (QPSK) and 16-level quadrature amplitude modulation (16-QAM). In addition, we set multiple antenna configurations for the AP: M∈{1,2,4}. For references, we plot the results under the assumption of independent Rayleigh fading channel.

During uplink transmissions (data transmission from the sensor to the AP), we considered a maximum ratio combining (MRC) scheme to improve receive diversity [20]. Similarly, we considered a space-time block coding (STBC) scheme to achieve transmit diversity during downlink transmissions (data transmission from the AP to the sensor) [21]. Especially, we considered the following STBC matrix for M=2 and M=4 [22,23]:

(8) $$\begin{array}{cc} C_{2}^{\mathrm{STBC}} & =\left[\begin{array}{cc} x_{1} & -x_{2}^{*}\\ x_{2} & x_{1}^{*} \end{array}\right],\\ C_{4}^{\mathrm{STBC}} & =\left[\begin{array}{cccc} x_{1} & -x_{2}^{*} & -x_{3}^{*} & 0\\ x_{2} & x_{1}^{*} & 0 & -x_{3}^{*}\\ x_{3} & 0 & x_{1}^{*} & x_{2}^{*}\\ 0 & x_{3} & -x_{2} & x_{1} \end{array}\right], \end{array}$$

where column and row indexes correspond to time-slot and transmit antenna, respectively, and $x_{n}$ denotes transmit symbol at *n* time-slot.

### 5.2. Simulation Results

**Figure 13:**
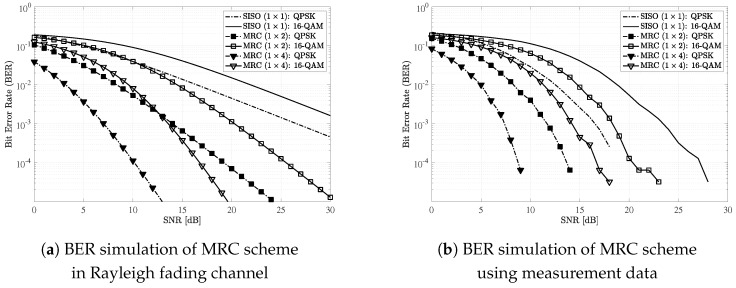
BER performance of MRC scheme in uplink transmission: Rayleigh fading vs. measurement data.

Figure 13 shows BER performance of the MRC scheme using two different datasets: independent Rayleigh channel coefficients and measurement data. Dashed-dot and solid lines indicate QPSK and 16-QAM results, respectively. In addition, lines without any marker indicate results for SISO (i.e., without MRC). Similarly, lines with square and inverted-triangle markers indicate 1×2 and 1×4 MRC results, respectively. When we considered Rayleigh channel, which reflects rich scattering environment based on multipath, the BER performance (Figure 13a) was clearly improved by using MRC schemes. However, when we considered the simulation results based on measurement data (Figure 13b), only power gain could be achieved by multiple receive antennas rather than diversity gain represented by the slope of the graph. As discussed in Remark 1, this was mainly due to the dominant effect of LOS in our measurement data.

**Figure 14:**
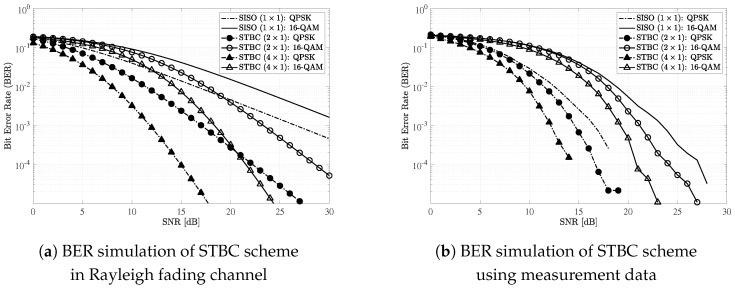
BER performance of STBC scheme in downlink transmission: Rayleigh fading vs. measurement data.

Figure 14 shows BER performance of the STBC scheme using two different datasets: independent Rayleigh channel coefficients and measurement data. Dashed-dot and solid lines indicate QPSK and 16-QAM results, respectively. In addition, lines without any marker indicate results for SISO (i.e., without STBC). Similarly, lines with square and inverted-triangle markers indicate 2×1 and 4×1 STBC results, respectively. Similar to the simulation presented in Figure 13, diversity gain was only achieved when we assumed Rayleigh fading channel. However, the results based on measurement data show better BER performance compared to the ones with M=1 and M=2 based on Rayleigh channel coefficients. In addition, the BER performance gap between modulations (QPSK and 16-QAM) increased when we considered the results based on measurement data, compared to the ones with Rayleigh fading channel.

**Remark** **3.** *Note that our observations in Figure 13b and Figure 14b such as no diversity gain, better BER performance, and bigger BER performance gap between modulations can be utilized to design wireless technologies applicable inside the aircraft.*

## 6. Conclusions

In this paper, we present the experimental results on wireless channels of the wireless avionics intra-communications (WAIC) in the real aircraft environment (KT-1 Woongbi). To transmit and receive wireless signals at the WAIC bands (4.2–4.4 GHz), we made monopole antennas. We adopted IEEE 802.11 physical-layer specification as a candidate techniques for high data-rate applications of the WAIC. We performed tests under several measurement points in the real aircraft and obtained channel measurement results. Based on the results, we analyzed power delay profile and the path loss characteristics of the WAIC. Furthermore, we simulated the BER performance using Rayleigh channel coefficients and our measurement data when we considered maximum ratio combining (MRC) and space-time block code (STBC) schemes in uplink and downlink transmissions, respectively. Our results emphasize the crucial importance of studying and applying wireless technologies in LOS dominant environment rather than simply assuming Rayleigh fading channel. In addition, the installation of relay nodes is highly required to guarantee the reliable wireless communication within the aircraft. An additional real-field experiment design using multiple relay nodes to wirelessly cover a big-sized aircraft remains as future work for in-depth WAIC feasibility test. The experimental results in this paper can be applied to design another aircraft.

## Figures and Tables

**Table 1:** OFDM parameters in IEEE 802.11 legacy mode [10].

Parameter	Value
Bandwidth	20 MHz
Operating bandwidth	16.6 MHz
Subcarrier spacing ($\Delta_{F}$)	31.25 KHz (20 MHz/64)
FFT period ($T_{\mathrm{FFT}}$)	$3.2\;\mu s\;(=1/\Delta_{F})$
Guard interval ($T_{\mathrm{GI}}$)	$0.8\;\mu s\;(=T_{\mathrm{FFT}}/4)$
OFDM symbol duration	$4.0\;\mu s\;(=T_{\mathrm{GI}}+T_{\mathrm{FFT}})$
Data rate	6/9/12/18/24/36/48/54 Mbps
Modulation	BPSK, QPSK, 16QAM, 64QAM
Coding rate	1/2, 2/3, 3/4
Total subcarriers	52 (Freq. index: −26 to +26)
Data subcarriers	48
Pilot subcarriers	4 (Freq. index: −21, −7, +7, +21)
DC subcarriers	Null (Freq. index: 0)

**Table 2:** Specification of KT-1 Woongbi [14].

Item	Description
Crew	two in tandem
Length	10.26 m
Wingspan	10.59 m
Height	3.68 m
Wing area	16.01 m^2^
Empty weight	1910 kg
Loaded weight	2540 kg
Max. takeoff weight	3331 kg
Powerplant	Pratt & Whitney Canada PT6A-62

**Table 3:** Classification of communication regions.

Classification	Locations
Region 1	P0, P1
Region 2	P0, P2, P3
Region 3	P4, P5
Region 4	P6, P7

**Figure A1:**
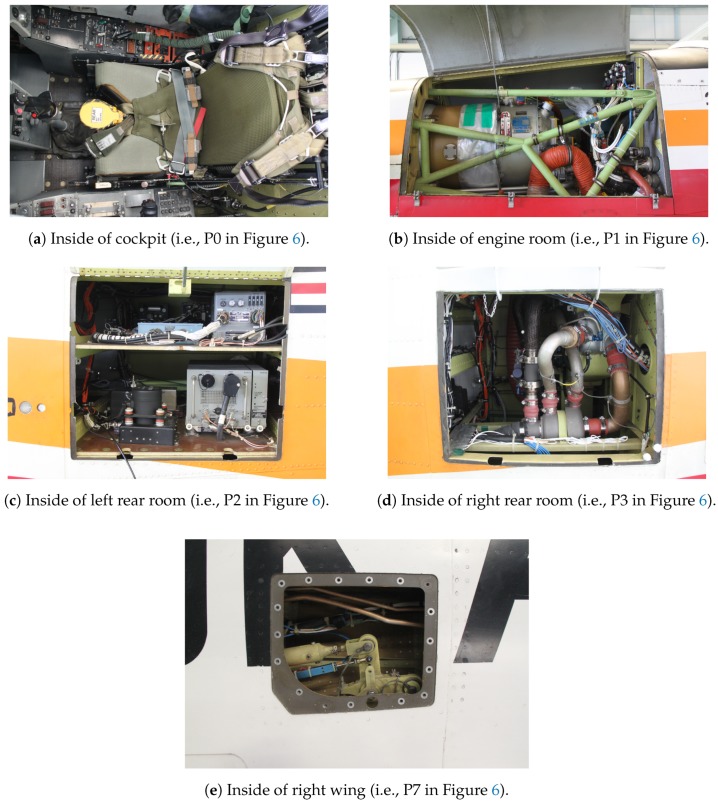
Inside photos of KT-1 Woongbi at several measurement points.

## Appendix A. Inside Photos of Measurement Points in the Feasibility Test

For clear understanding about the details of the experimental setting, we provide inside photos of KT-1 Woongbi at each measurement point described in Figure 6 if available. Figure A1 shows inside of measurement points in KT-1 Woongbi. As we expected, electrical devices, mechanical components, and cables are filled inside.
